# A divergent asymmetric approach to aza-spiropyran derivative and (1*S*,8a*R*)-1-hydroxyindolizidine

**DOI:** 10.1186/1860-5397-3-41

**Published:** 2007-11-08

**Authors:** Jian-Feng Zheng, Wen Chen, Su-Yu Huang, Jian-Liang Ye, Pei-Qiang Huang

**Affiliations:** 1 Department of Chemistry and Key Laboratory for Chemical Biology of Fujian Province, College of Chemistry and Chemical Engineering, Xiamen University, Xiamen, Fujian 361005, P. R. China

## Abstract

**Background:**

Spiroketals and the corresponding aza-spiroketals are the structural features found in a number of bioactive natural products, and in compounds possessing photochromic properties for use in the area of photochemical erasable memory, self-development photography, actinometry, displays, filters, lenses of variable optical density, and photomechanical biomaterials etc. And (1*R*,8a*S*)-1-hydroxyindolizidine (**3**) has been postulated to be a biosynthetic precursor of hydroxylated indolizidines such as (+)-lentiginosine **1**, (−)-2-epilentiginosine **2** and (−)-swainsonine, which are potentially useful antimetastasis drugs for the treatment of cancer. In continuation of a project aimed at the development of enantiomeric malimide-based synthetic methodology, we now report a divergent, concise and highly diastereoselective approach for the asymmetric syntheses of an aza-spiropyran derivative **7** and (1*S*,8a*R*)-1-hydroxyindolizidine (*ent-***3**).

**Results:**

The synthesis of aza-spiropyran **7** started from the Grignard addition of malimide **4**. Treatment of the THP-protected 4-hydroxybutyl magnesium bromide with malimide **4** at −20°C afforded *N*,*O*-acetal **5a** as an epimeric mixture in a combined yield of 89%. Subjection of the diastereomeric mixture of *N*,*O*-acetal **5a** to acidic conditions for 0.5 h resulted in the formation of the desired functionalized aza-spiropyran **7** as a single diastereomer in quantitative yield. The stereochemistry of the aza-spiropyran **7** was determined by NOESY experiment. For the synthesis of *ent*-**3**, aza-spiropyran **7**, or more conveniently, *N*,*O*-acetal **5a**, was converted to lactam **6a** under standard reductive dehydroxylation conditions in 78% or 77% yield. Reduction of lactam **6a** with borane-dimethylsulfide provided pyrrolidine **8** in 95% yield. Compound **8** was then converted to 1-hydroxyindolizidine *ent*-**3** via a four-step procedure, namely, *N*-debenzylation/*O*-mesylation/Boc-cleavage/cyclization, and *O*-debenzylation. Alternatively, amino alcohol **8** was mesylated and the resultant mesylate **12** was subjected to hydrogenolytic conditions, which gave (1*S*,8a*R*)-1-hydroxyindolizidine (*ent*-**3**) in 60% overall yield from **8**.

**Conclusion:**

By the reaction of functionalized Grignard reagent with protected (*S*)-malimide, either aza-spiropyran or (1*S*,8a*R*)-1-hydroxyindolizidine skeleton could be constructed in a concise and selective manner. The results presented herein constitute an important extension of our malimide-based synthetic methodology.

## Background

**Scheme 1:**
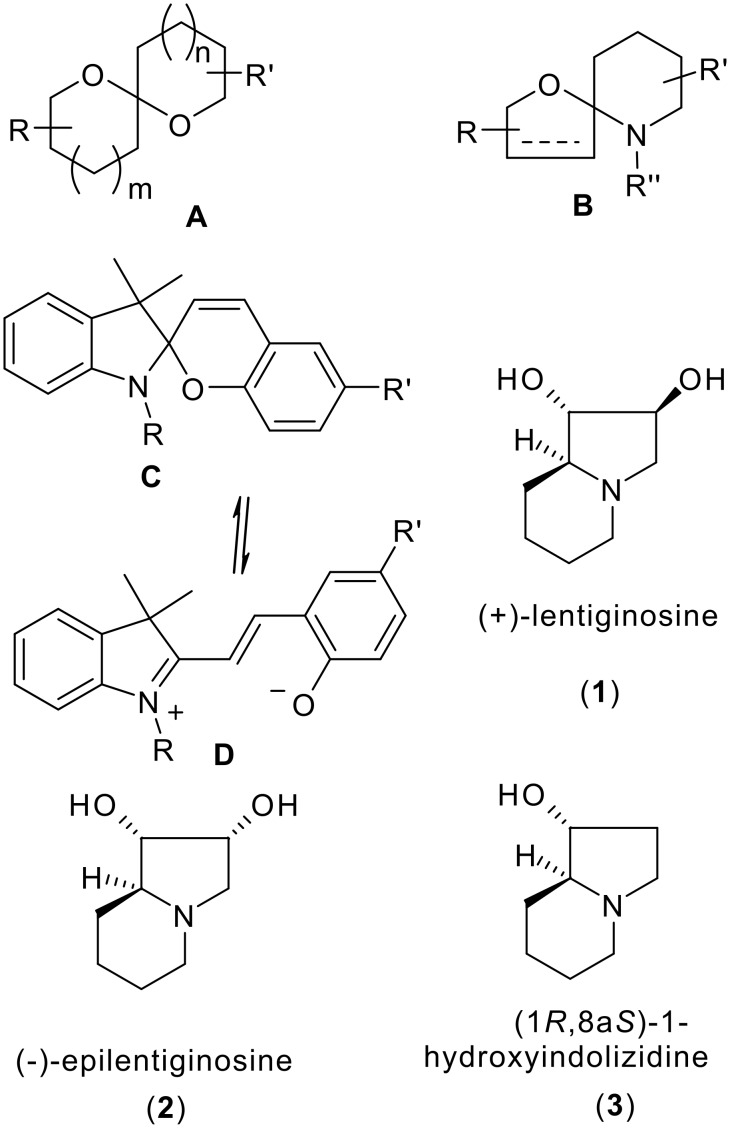
The skeletons of useful aza-spiroketals and some naturally occurring hydroxylated indolizidines.

Spiroketals of general structure **A** (Scheme 1) constitute key structural features of a number of bioactive natural products isolated from insects, microbes, fungi, plants or marine organisms. [1–3] The corresponding aza-spiroketal (cf: general structure **B**) containing natural products, while less common, are also found in plants, shellfish and microbes.[4–5] For example, pandamarilactone-1 and pandamarine were isolated from the leaves of *Pandanus amaryllifolius*;[6] solasodine and its derivatives were isolated from *Solanum umbelliferum*, which exhibited significant activity toward DNA repair-deficient yeast mutants;[7] azaspiracids are marine phycotoxins isolated from cultivated mussels in Killary harbor, Ireland;[8] and chlorofusin A is a novel fungal metabolite showing the potential as a lead in cancer therapy.[9] In addition, aza-spiropyrans **C**, being able to equilibrate with the corresponding non-spiro analogue **D**, is a well known class of compounds possessing photochromic properties for use in the area of photochemical erasable memory,[10] and also found applications as self-development photography, actinometry, displays, filters, lenses of variable optical density,[11] and photomechanical biomaterials etc.[12]

On the other hand, hydroxylated indolizidines [13–20] such as castanospermine, (−)-swainsonine, (+)-lentiginosine (**1**) [21–23] and (−)-2-epilentiginosine (**2**) [21–26] constitute a class of azasugars showing potent and selective glycosidase inhibitory activities. [13–20] (1*R*,8a*S*)-1-Hydroxyindolizidine **3** has been postulated as a biosynthetic precursor [21–26] of (+)-lentiginosine (**1**), (−)-2-epilentiginosine (**2**) and (−)-swainsonine, a potentially useful antimetastasis drug for the treatment of cancer.[15] In addition, these molecules serve as platforms for testing synthetic strategies, and several asymmetric syntheses of both enantiomers of 1-hydroxyindolizidine (**3**) have been reported. [27–34]In continuation of our efforts in the development of enantiomeric malimide-based synthetic methodologies, [35–38] we now report concise and highly diastereoselective syntheses of an aza-spiropyran derivative **7** and (1*S*,8a*R*)-1-hydroxyindolizidine (*ent*-**3**).

## Results and discussion

**Scheme 2:**
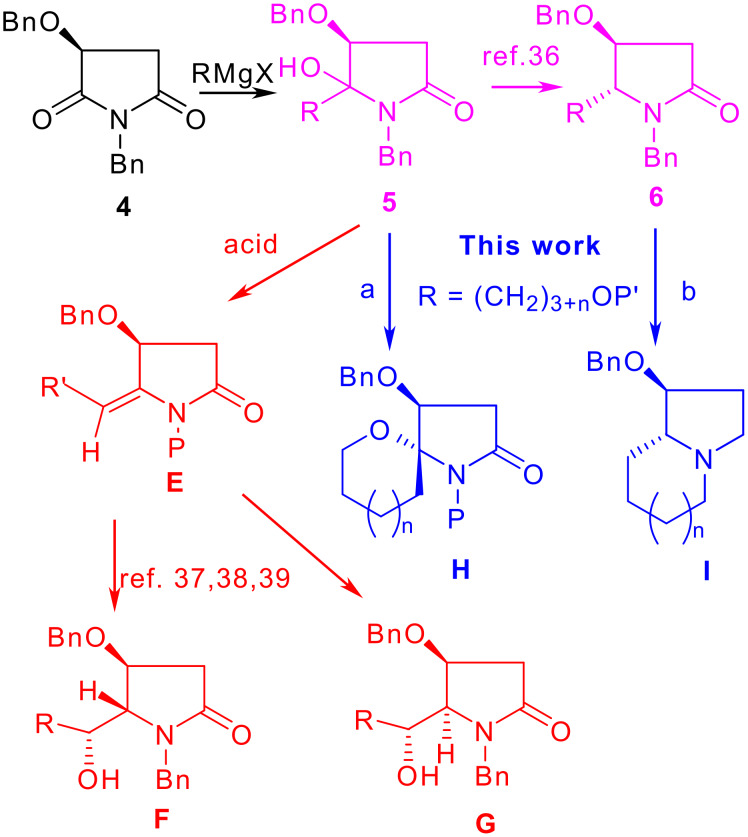
Synthetic strategy based on *N*,*O*-dibenzylmalimide (**4**).

Previously, we have shown that the addition of Grignard reagents to *N*,*O*-dibenzyl malimide (**4**) leads to *N*,*O*-acetals **5** in high regioselectivity (Scheme 2), and the subsequent reductive dehydroxylation gives **6** in high *trans*-diastereoselectivity.[35] On the other hand, treatment of *N*,*O*-acteals **5** with an acid furnished enamides **E**, which can be transformed stereoselectively to either hydroxylactams **F** or **G** under appropriate conditions. [36–38] It was envisioned that if a C_4_-bifunctional Grignard reagent was used, both aza-spiroketal **H** (such as aza-spiropyran, n = 1, path a) and indolizidine ring systems **I** (path b) could be obtained.

**Scheme 3:**
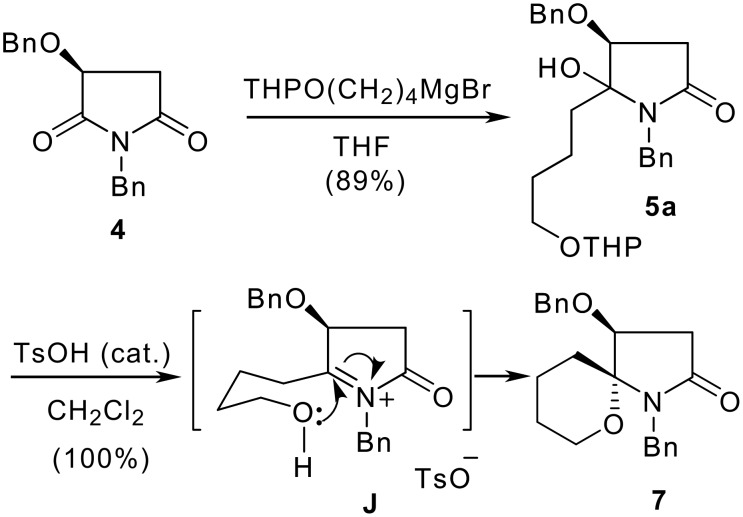
Stereoselectivity synthesis of aza-spiropyran **7**.

The synthesis of aza-spiropyran **7** started from the Grignard addition of malimide **4**. Treatment of the THP-protected 4-hydroxybutyl magnesium bromide with malimide **4** at −20°C for 2.5 h afforded *N*,*O*-acetal **5a** as an epimeric mixture in 7:1 ratio and with a combined yield of 89% (Scheme 3). If the reaction was allowed to stir at room temperature overnight, the diastereomeric ratio was inversed to 1: 1.8. Subjection of the diastereomeric mixture of the *N*,*O*-acetal **5a** to acidic conditions [TsOH (cat.)/CH_2_Cl_2_, r.t.] for 0.5 h resulted in the formation of the desired functionalized aza-spiropyran derivative **7** as a single diastereomer in quantitative yield. The result means that a tandem dehydration-THP cleavage-intramolecular nucleophilic addition occurred. When the stirring was prolonged to 2 h, about 5% of another epimer (no shown) was also formed according to the ^1^H NMR analysis.

**Figure 1:**
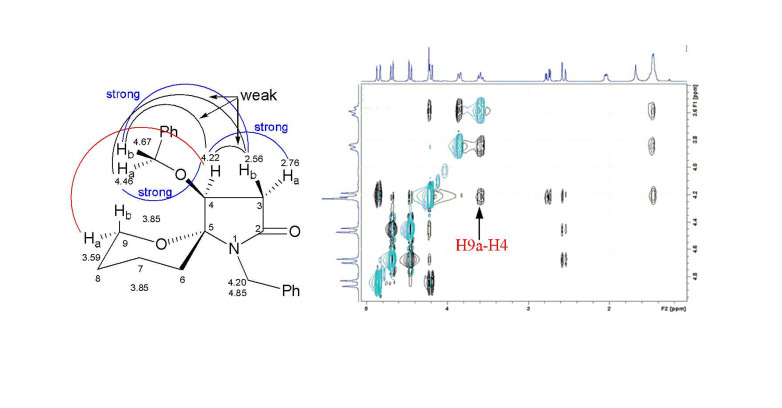
The observed NOE correlations (in part) and the region expanded NOESY spectrum of compound **7**.

The stereochemistry of the aza-spiropyran **7** was determined on the basis of the NMR analysis. This was done firstly by a ^1^H-^1^H COSY experiment to confirm the proton assignments, and then by NOESY experiment. As shown in Figure 1, the strong NOE correlation of H-9a (δ_H_ 3.59) and H-4 (δ_H_ 4.22) indicates clearly O_4_/O_5_-*trans* relationship in compound **7**.

These findings are surprising comparing with our recent observations. In our previous investigations, it was observed that the treatment of *N*,*O*-acetals **5** with an acid leads to the dehydration products **E** (Scheme 2), and the two diastereomers of **5** shows different reactivities towards the acid-promoted dehydration. [36–38] The *trans*-diastereomer reacts much more slower than the *cis*-diastereomer, and some un-reacted *trans*-epimer was always recovered even starting with a pure *cis-*diastereomer. In the present study, not only both two diastereomers have been completely converted to the aza-spiropyran **7**, what is equally surprising is that no dehydration product was observed under acidic conditions!

**Scheme 4:**
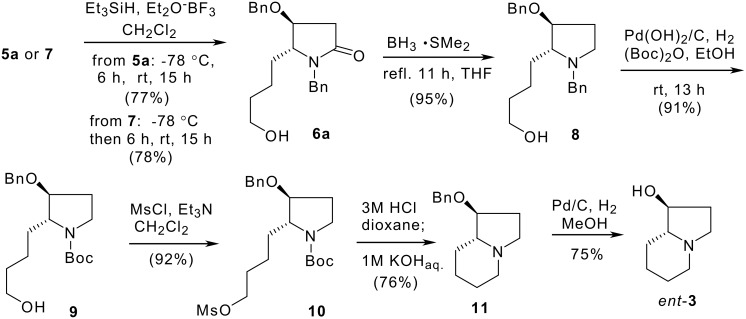
Stereoselective synthesis of (1*S*,8a*R*)-1-hydroxyindolizidine (*ent*-**3**).

For the synthesis of *ent*-**3**, aza-spiropyran **7**, a cyclic *N*,*O*-acetal, was converted to lactam **6a** under standard reductive dehydroxylation conditions (Et_3_SiH, BF_3_·OEt_2_, −78°C, 6 h; warm-up, yield: 78%) (Scheme 4). Under the same conditions, *N*,*O*-acetal **5a** was converted to lactam **6a** in 77% yield. It was observed that during the reaction of **5a**, **7** was first formed as an intermediate after the addition of Et_3_SiH and BF_3_·OEt_2_, and stirring for 1 hour.

Reduction of lactam **6a** with borane-dimethylsulfide provided pyrrolidine derivative **8** in 95% yield. Compound **8** was then converted to (1*S*,8a*R*)-1-hydroxyindolizidine (*ent*-**3**) {[α]_D_^27^ +50 (*c* 0.90, EtOH); lit.[29] [α]_D_ +51.0 (*c* 0.54, EtOH); lit.[32] −49.7 (*c* 0.95, EtOH) for the antipode} via a four-step procedure, namely, one-pot *N*-debenzylation-*N*-Boc formation/*O*-mesylation/Boc-cleavage/cyclication, and *O*-debenzylation.

**Scheme 5:**
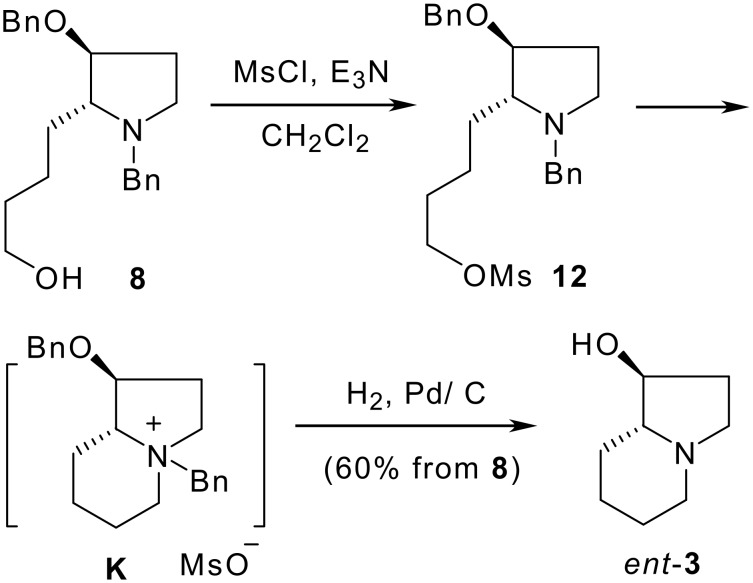
One-pot synthesis of *ent*-**3** from amino alcohol **8**.

In searching for a more concise method, amino alcohol **8** was mesylated (MsCl, NEt_3_, 0 °C) and the resultant labile mesylate **12** was subjected to catalytic hydrogenolysis (H_2_, l atm, 10% Pd/C, r.t.), which gave (1*S*,8a*R*)-1-hydroxyindolizidine (*ent*-**3**) in 60% overall yield from **8** (Scheme 5).[39–40] The one-pot *N*,*O*-bis-debenzylation and cyclization of mesylate **12** deserves comment. Because the *N*-debenzylation generally required longer reaction time,[41] or using of Pearlman's catalyst (cf. Scheme 4). The easy debenzylation of **12** allows assuming that an intramolecular substitution occurred firstly, and the formation of the quaternary ammonium salt **K** [40] then favors the reductive debenzylation. This mechanism is supported by the following observations. First, in a similar case, Thompson et al observed that the formation of a mesylate resulted in spontaneous quarternization leading to the bicyclic indolizidine.[40] Second, we have also observed that the tosylate of **8** is too labile to be isolated, and mesylate **12** decomposed upon flash column chromatography on silica gel, which are due to the spontaneous formation of a polar quaternary ammonium salt. In addition, the presence of the *O*-benzyl group in **K** is an assumption based on our previous observation on a similar case.[42]

## Conclusion

In summary, we have demonstrated that by the reaction of functionalized Grignard reagent with the protected (*S*)-malimide **4**, either aza-spiropyran derivative **7** or (1*S*,8a*R*)-1-hydroxyindolizidine skeleton (*ent*-**3**) can be constructed in a concise and selective manner. It is worthy of mention that in addition to the reductive dehydroxylation leading to 2-pyrrolidinones **6**, and the acid-promoted dehydration leading to (*E*)-enamides **E** (and then **F**, **G**), acid treatment of the *N*,*O*-acetal **5a** could provide, chemoselectively and quantitatively, the aza-spiropyran ring system **7**. The results presented herein constitute a valuable extension of our malimides-based synthetic methodology.

See Supporting Information File 1 for full experimental procedures and characterization data of the synthesized compounds.

## Supporting Information

File 1 Experimental. Experimental procedures for the synthesis of all compounds described, and characterization data for the synthesized compounds.
