# Supplementary material for: A divergent asymmetric approach to aza-spiropyran derivative and (1S,8aR)-1-hydroxyindolizidine
Source: Beilstein J Org Chem. 2007 Nov 8;3:41. doi: 10.1186/1860-5397-3-41 (PMC2200668; doi:10.1186/1860-5397-3-41)
Supplement: File 1 — Experimental. Experimental procedures for the synthesis of all compounds described, and characterization data for the synthesized compounds. [file Beilstein_J_Org_Chem-03-41-s001.doc]

Supporting Information for

A divergent asymmetric approach to aza-spiropyran derivative and (1*S*,8a*R*)-1-hydroxyindolizidine

Jian-Feng Zheng, Wen Chen, Su-Yu Huang, Jian-Liang Ye and Pei-Qiang Huang*

Address: Department of Chemistry and Key Laboratory for Chemical Biology of Fujian Province, College of Chemistry and Chemical Engineering, Xiamen University,

Xiamen, Fujian 361005, P. R. China

Experimental

**General.** Melting points were determined on a Yanaco MP-500 melting point apparatus and are uncorrected. Infrared spectra were measured with a Nicolet Avatar 330 FT-IR spectrometer using film KBr pellet technique. ^1^H NMR spectra were recorded in CDCl_3_ on a Bruker AV400 or a Varian unity+ 500 spectrometer with tetramethylsilane as an internal standard. Chemical shifts are expressed in δ (ppm) units downfield from TMS. Mass spectra were recorded by a Bruker Dalton Esquire 3000 plus LC-MS apparatus. Optical rotations were measured with a Perkin-Elmer 341 automatic polarimeter. Flash column chromatography was carried out on silica gel (300-400 mesh). THF was distilled over sodium. Dichloromethane was distilled over P_2_O_5_. Petroleum ether (60-90 °C) is abbreviated as P. E.

**(4*S,*5*R/S*)-1-Benzyl-4-(benzyloxy)-5-hydroxy-5-(4-(tetrahydro-2H-pyran-2-yloxy)butyl)pyrrolidin-2-one (5a)**

To a stirring cooled (–17 ^o^C) THF solution (13.4 mL) of (*S*)-*N*,*O*-dibenzylmalimide **4** [35] (0.810 g, 2.74 mmol) was added dropwise a 0.95 M THF solution of freshly prepared 4-(2-(tetrahydro-2*H*-pyran)-oxy)butyl magnesium bromide (8.5 mL, 8.2 mmol) under an atmosphere of N_2_. After stirring at −10 ~ −15 ^o^C for 2.5 hours, the reaction was quenched with water (10 mL). After diluting with diethyl ether (5 mL), the mixture was poured into a separatory funnel and the organic layer was separated. The aqueous phase was extracted with ether (3×8 mL). The combined organic phases were washed with brine, dried over anhydrous Na_2_SO_4_, filtered and concentrated under reduced pressure. Flash chromatographic purification (eluent: ethyl acetate: P. E. = 1:2) of the residue provided **5a** (1.11 g, 89%) as a mixture of two diastereomers, which was used in the subsequent step without further separation.

**(4*S*,5*R*)-1-Benzyl-2-oxo-4-(benzyloxy)-1-aza-6-oxaspiro [4.5] decane (7)**

To a solution of **5a** (196 mg, 0.433 mmol) in CH_2_Cl_2_ (1.8 mL) was added *p*-toluenesulfonic acid (19 mg, 0.11 mmol). After stirring for 0.5 hours at 0 ^o^C, the mixture was concentrated under reduced pressure and purified by flash chromatography on silica gel (eluent: ethyl acetate: P. E. = 1: 3) to provide **7** (152 mg, 100%) as a colorless oil. [α]_D_^20^ +82.0 (*c* 0.70, CHCl_3_). ^1^H NMR (400 MHz, CDCl_3_) δ: 1.30-1.60 (m, 4H, CH_2_), 1.60-1.75 (m, 1H, CH_2_), 1.95-2.10 (m, 1H, CH_2_), 2.56 (d, *J* = 17.15 Hz, 1H, CH_2_C=O), 2.76 (dd, *J* = 5.55, 17.15 Hz, 1H, CH_2_C=O), 3.60 (dm, *J* = 11.26 Hz, 1H, OCH_2_), 3.85 (dm, *J* = 11.26 Hz, 1H, OCH_2_), 4.21 (d, *J* = 16.30 Hz, 1H, PhCH_2_N), 4.22 (d, *J* = 5.55 Hz, 1H, OCH), 4.46 (d, *J* =11.69 Hz, 1H, PhCH_2_O), 4.69 (d, *J* =11.69 Hz, 1H, PhCH_2_O), 4.85 (d, *J* = 16.30 Hz, 1H, PhCH_2_N), 7.05-7.40 (m, 10H, Ph-H) ppm; ^13^C NMR (100 MHz, CDCl_3_) δ: 20.01 (1C), 24.70 (1C), 27.99 (1C), 34.90 (1C), 42.57 (1C), 64.84 (1C), 71.07 (1C), 72.84 (1C), 95.11 (1C), 126.67 (2C), 126.73 (1C), 127.68 (2C), 127.88 (1C), 128.28 (2C), 128.42 (2C), 137.36 (1C), 138.47 (1C), 174.63 (1×C=O) ppm; MS (ESI): 352 (M+H^+^, 100).

**(4*S*,5*R*)-1-Benzyl-4-(benzyloxy)-5-(4-hydroxybutyl)pyrrolidin-2-one (6a)**

To a cooled (−78 ^o^C) dichloromethane solution (22 mL) of the diastereomeric mixture of **5a** (1.10 g, 2.30 mmol) were added successively Et_3_SiH (3.65 mL, 23.0 mmol) and BF_3_•OEt_2_ (1.18 mL, 9.30 mmol). The resulting mixture was stirred at −78 ^o^C for 6 hours, and then allowed to warm up. After stirring at room temperature for 48 hours, the mixture was quenched by adding a saturated aqueous solution of NaHCO_3_ (15 mL) at 0 ^o^C. After being diluted with dichloromethane (10 mL), the mixture was poured into a separatory funnel. The organic layer was separated, and the aqueous phase was extracted with CH_2_Cl_2_ (3×10 mL). The combined organic phases were washed with brine, dried over anhydrous Na_2_SO_4_, filtered and concentrated under reduced pressure. After flash chromatographic purification (eluent: ethyl acetate: P. E. = 1:1), pyrrolidinone **6a** was obtained as a colorless solid (0.62 g, 77 %). M. p. = 71-73 °C. [α]_D_^20^ +38.6 (*c* 1.07, CHCl_3_). IR (film) ν_max_: 3412, 3062, 3030, 2929, 2860, 1674, 1496, 1452, 1357, 1307, 1259, 1069, 1028 cm^-1^; ^1^H NMR (500 MHz, CDCl_3_) δ 1.20-1.80 (m, 6H, CH_2_), 2.55 (dd, *J* = 2.02, 17.57 Hz, 1H, CH_2_C=O), 2.74 (dd, *J* = 6.02, 17.57 Hz, 1H, CH_2_C=O), 3.49 (m, 1H, NCH), 3.57 (t, *J* = 6.32 Hz, 2H, OCH_2_), 3.89 (dm, *J* = 6.41 Hz, 1H, OCH), 3.95 (d, *J* = 15.20 Hz, 1H, PhCH_2_N), 4.40 (d, *J* = 11.81 Hz, 1H, PhCH_2_O), 4.47 (d, *J* = 11.81 Hz, 1H, PhCH_2_O), 5.05 (d, *J* = 15.20 Hz, 1H, PhCH_2_N), 7.05-7.55 (m, 10H, Ph-H) ppm; ^13^C NMR (125 MHz, CDCl_3_) δ: 20.99 (1C), 30.33 (1C), 32.35 (1C), 37.29 (1C), 43.99 (1C), 62.13 (1C), 63.13 (1C), 70.45 (1C), 75.64 (1C), 127.40 (2C), 127.57 (2C), 127.77 (2C), 128.37 (2C), 128.59 (2C), 136.16 (1C), 137.42 (1C), 172.69 (1×C=O) ppm; HRMS calcd for [C_22_H_27_NO_3_+H]^+^: 354.2064; found: 354.2060.

**4-[(2*R*,3*S*)-1-Benzyl-3-(benzyloxy)pyrrolidin-2-yl]butan-1-ol (8)**

To a cooled (–15 ^o^C) solution of **6a** (416 mg, 1.18 mmol) in anhydrous THF (15 mL) was added dropwise BH_3_•SMe_2_ (0.86 mL, 9 mmol) over a period of 3 min. After stirring for 1 hour at room temperature, the reaction was heated to 66 ^o^C and stirred for 11 hours. The resulting mixture was chilled with an ice bath, and then quenched with H_2_O (10 mL). The resultant mixture was extracted with CH_2_Cl_2_ (3×10 mL). The combined organic phases were dried over anhydrous Na_2_SO_4_, filtered and concentrated under reduced pressure. The residue was purified by flash chromatography on silica gel (eluent: ethyl acetate: P. E. = 1: 1) to provide **8** (380 mg, 95%) as a colorless oil. [α]_D_^20^ −24 (*c* 1.08, CHCl_3_). IR (film) ν_max_: 3389, 3062, 3028, 1495, 1454, 1367, 1096, 1064, 1028 cm^-1^; ^1^H NMR (500 MHz, CDCl_3_) δ: 1.30-1.66 (m, 5H, CH_2_), 1.72-1.90 (m, 3H, CH_2_), 2.42 (ddd, *J* = 7.06, 9.13, 10.59 Hz, 1H, NCH), 2.54 (m, 1H, NCH_2_), 2.85 (m, 1H, NCH_2_), 3.33 (d, *J* = 12.90 Hz, 1H, PhCH_2_N), 3.57 (t, *J* = 6.52 Hz, 2H, OCH_2_), 3.77 (m, 1H, OCH), 3.96 (d, *J* = 12.90 Hz, 1H, PhCH_2_N), 4.42 (d, *J* = 11.81 Hz, 1H, PhCH_2_O), 4.53 (d, *J* = 11.81 Hz, 1H, PhCH_2_O), 7.05-7.40 (m, 10H, Ph-H) ppm; ^13^C NMR (125 MHz, CDCl_3_) δ: 20.99 (1C), 30.33 (1C), 32.35 (1C), 37.29 (1C), 43.99 (1C), 62.13 (1C), 63.13 (1C), 70.45 (1C), 75.64 (1C), 127.40 (1C), 127.57 (2C), 127.77 (2C), 128.37 (2C), 128.59 (2C), 136.16 (1C), 137.42 (1C), 172.69 (1C) ppm; HRMS calcd for [C_22_H_27_NO_2_+H]^+^: 340.2271; found: 340.2271.

**(2*R*,3S)-*tert*-Butyl 3-(benzyloxy)-2-(4-hydroxybutyl)pyrrolidine-1-carboxylate (9)**

To a mixture of **8** (280 mg, 0.83 mmol) and 20% Pd(OH)_2_/C (50 mg) were added ethanol (6 mL) and di-*tert*-butyldicarbonate (0.54 mL, 2.48 mmol). The mixture was stirred at room temperature and under an atmosphere of H_2_ for 13 hours. The resulting mixture was filtered through Celite and the filtrate was concentrated. The residue was purified by flash chromatography on silica gel (eluent: ethyl acetate: P. E. = 1: 1) to provide **9** (250 mg, 91%) as a colorless oil. [α]_D_^20^ –17.6 (*c* 1.20, CHCl_3_). IR (film) ν_max_: 3434, 3063, 3030, 2975, 2929, 2860, 1690, 1672, 1454, 1398, 1364, 1254, 1172, 1097, 1065, 1028 cm^-1^; ^1^H NMR (500 MHz, CDCl_3_, rotamers) δ: 1.20-1.75 (m. 5H, CH_2_), 1.45 (s, 9H, CH_3_), 1.85-2.10 (m, 3H, CH_2_), 3.33-3.57 (m, 2H), 3.62 (t, *J* = 6.12 Hz, 2H, CH_2_O), 3.75 (m, 0.5H), 3.81 (dm, *J* = 9.51 Hz, 1H), 3.91 (m, 0.5H), 4.51 (m, 2H, PhCH_2_O) ppm; ^13^C NMR (125 MHz, CDCl_3_, rotamers) δ: 22.69, 28.58, 29.68, 32.63, 33.24, 44.26, 44.71, 62.55, 62.99, 70.48, 79.30, 81.31, 82.27, 127.68, 128.41, 138.26, 154.85, 155.09 ppm; HRMS calcd for [C_20_H_31_NO_4_+H]^+^: 350.2327; found: 350.2328.

**(2*R*,3*S*)-*tert*-Butyl 3-(benzyloxy)-2-[4-(methylsulfonyloxy)butyl]pyrrolidine-1-carboxylate (10)**

To a cooled (0 ^o^C) CH_2_Cl_2_ solution (3.4 mL) of **9** (190 mg, 0.54 mmol) were added successively Et_3_N (0.12 mL, 0.82 mmol) and MsCl (0.054 mL, 0.70 mmol). After stirring for 12 hours, the mixture was quenched by addition of a saturated aqueous solution of NH_4_Cl (2 mL) and H_2_O (1.2 mL). The aqueous layer was separated and extracted with CH_2_Cl_2_ (3×5 mL). The combined organic phases were washed with brine (2 mL), and then dried over anhydrous Na_2_SO_4_, filtered and concentrated under reduced pressure. The residue was purified by flash chromatography on silica gel (eluent: ethyl acetate: P. E. = 1: 2) to provide **10** (214 mg, 92%) as a colorless oil. [α]_D_^20^ –14.5 (*c* 0.98, CHCl_3_). IR (film) ν_max_: 2973, 2930, 2865, 1689, 1454, 1396, 1355, 1174, 1108, 1065 cm^-1^; ^1^H NMR (500 MHz, CDCl_3_, rotamers) δ: 1.20- 1.80 (m, 6H, CH_2_), 1.45 (s, 9H, CH_3_), 2.00 (m, 2H, CH_2_), 2.95 (s, 3H, SCH_3_), 3.39 (m, 1H), 3.43-3.62 (m, 1H), 3.70-3.92 (m, 2H), 4.21 (t, *J* = 6.37 Hz, 2H, OCH_2_), 4.52 (m, 2H, PhCH_2_O) ppm; ^13^C NMR (125 MHz, CDCl_3_, rotamers) δ: 22.34, 28.49, 28.80, 29.55, 32.18, 32.85, 37.29, 44.20, 44.61, 62.36, 62.68, 69.57, 69.82, 70.41, 79.13, 79.33, 81.05, 82.15, 127.59, 128.42, 138.07, 154.83 ppm; HRMS calcd for [C_21_H_33_NO_6_S+H]^+^: 428.2339; found: 428.2343.

**(1*S*,8a*R*)-1-(Benzyloxy)-octahydroindolizine (11)**

To a dioxane solution (1.24 mL) of mesylate **10** (80 mg, 0.19 mmol) was added a solution of 3N HCl (1.24 mL). After stirring for 24 hours, the resulting mixture was concentrated under reduced pressure. The residue was taken with water (2 mL) and washed with CH_2_Cl_2_ (3×3 mL). The combined organic layers were backed-extracted with water (3 mL). The combined aqueous fractions were adjusted to pH 11 with a solution of 1N NaOH, and stirred for 3 hours. The resulting mixture was extracted with CH_2_Cl_2_ (3×3 mL). The combined organic extracts were dried over anhydrous Na_2_SO_4_, filtered, and concentrated under reduced pressure. The residue was purified by flash chromatography on silica gel (eluent: ethyl acetate: P. E.: NH_4_OH = 3: 1: 0.04) to provide **11** (32 mg, 76%) as a colorless oil: [α]_D_^20^ +69.0 (*c* 1.24, CHCl_3_). IR (film) ν_max_: 3030, 2931, 2853, 2785, 1549, 1496, 1453, 1327, 1259, 1093, 1027 cm^-1^; ^1^H NMR (500 MHz, CDCl_3_) δ: 1.18-1.34 (m, 2H, CH_2_), 1.51 (m, 1H, CH_2_), 1.58-1.70 (m, 2H, CH_2_), 1.80 (m, 1H, CH_2_), 1.90 (m, 1H, CH_2_), 1.98-2.08 (m, 2H, NCH_2_, CH_2_), 2.30 (ddd, *J* = 8.9, 13.3, 17.8 Hz, 1H, NCH_2_), 2.98 (dt, *J* = 1.38, 8.75 Hz, 1H, NCH), 3.02 (m, 1H, NCH_2_), 3.72 (ddd, *J* = 3.99, 7.48, 8.75 Hz, 1H, OCH), 4.47 (d, *J* = 11.67 Hz, 1H, PhCH_2_O), 4.57 (d, *J* = 11.67 Hz, 1H, PhCH_2_O) ppm; ^13^C NMR (125 MHz, CDCl_3_) δ: 24.27, 25.31, 29.10, 29.77, 52.90, 53.49, 69.39, 71.97, 83.44, 127.55, 127.75, 127.86, 128.43, 128.54, 138.57 ppm; HRMS calcd for [C_15_H_21_NO+H]^+^: 232.1696; found: 232.1691.

**(1*S*,8a*R*)-Octahydroindolizin-1-ol (*ent*-3) (method 1, Scheme 4)**

A mixture of **11** (27 mg, 0.117 mmol), 10 % Pd/C (30 mg) and methanol (2 mL) was stirred at room temperature and under an atmosphere of H_2_ for 1 day. The mixture was filtered through Celite. To the filtrate was added 10 % Pd/C (30 mg), and the mixture was stirred at room temperature and under an atmosphere of H_2_ for 1 day. After being filtered through Celite, and concentrated under reduced pressure, the residue was purified by flash chromatography on silica gel (eluent: CH_2_Cl_2_: MeOH: NH_4_OH = 15: 1: 0.16) to give *ent-***3** (12 mg, 75%) as a pale yellow oil: [α]_D_^20^ +50 (*c*0.90, EtOH) {lit.^29^ [α]_D_^20^ +51 (*c* 0.54, EtOH)}. IR (film) ν_max_: 3357, 2928, 2853, 2792, 1671, 1442, 1326, 1259, 1143, 1101, 1072, 1031 cm^-1^; ^1^H NMR (500 MHz, CDCl_3_) δ: 1.26 (m, 2H, CH_2_), 1.50-1.62 (m, 2H, CH_2_), 1.65 (m, 1H, CH_2_), 1.84 (m, 1H, CH_2_), 1.90 (tm, *J* = 7.36 Hz, 1H, CH_2_), 2.00 (m, 1H, CH_2_), 2.14 (dt, *J* = 11.74, 3.06 Hz, 1H, NCH_2_), 2.28 (ddd, *J* = 8.75, 13.5, 17.80 Hz, 1H, NCH_2_), 2.46 (dd, *J* = 9.06, 17.80 Hz, 1H, NCH_2_), 3.03 (dt, *J* = 2.45, 9.06 Hz, 1H, NCH), 3.06 (m, 1H, NCH_2_), 3.96 (ddd, *J* = 4.60, 7.25, 9.05 Hz, 1H, CHO) ppm; ^13^C NMR (125 MHz, CDCl_3_) δ: 23.83, 24.51, 28.26, 31.71, 52.19, 52.93, 70.88, 75.92 ppm; HRMS calcd for [C_8_H_15_NO+H]^+^: 142.1226; found: 142.1226.

**(1*S*,8a*R*)-Octahydroindolizin-1-ol (*ent*-3) (method 2, Scheme 5)**

To a cooled (0 ^o^C) CH_2_Cl_2_ solution (3 mL) of **8** (110 mg, 0.32 mmol) were added successively Et_3_N (0.06 mL, 0.4 mmol) and MsCl (0.03 mL, 0.39 mmol). After stirring for 12 hours, the resulting mixture was concentrated under reduced pressure. To the resulting residue were added successively 10 % Pd/C (100 mg), ethanol (7 mL) and concentrated HCl (0.1 mL, 1.3 mmol). The mixture was stirred at room temperature and under an atmosphere of H_2_ for 5 days. The mixture was filtered through Celite. The solution was concentrated under reduced pressure, and the residue was purified by flash chromatography on silica gel (eluent: CH_2_Cl_2_: MeOH: NH_4_OH = 15: 1: 0.16) to provide *ent-***3** (24 mg, 60%) as a pale yellow oil. [α]_D_^20^ +50 (*c* 0.70, EtOH) {lit.^29^ [α]_D_^28^ +51 (*c* 0.54, EtOH)}.
